# Artificial intelligence to predict the BRAF^V600E^ mutation in patients with thyroid cancer

**DOI:** 10.1371/journal.pone.0242806

**Published:** 2020-11-25

**Authors:** Jiyoung Yoon, Eunjung Lee, Ja Seung Koo, Jung Hyun Yoon, Kee-Hyun Nam, Jandee Lee, Young Suk Jo, Hee Jung Moon, Vivian Youngjean Park, Jin Young Kwak

**Affiliations:** 1 Department of Radiology, Severance Hospital, Research Institute of Radiological Science, Yonsei University, College of Medicine, Seoul, South Korea; 2 Department of Computational Science and Engineering, Yonsei University, Seoul, South Korea; 3 Department of Pathology, Severance Hospital, Yonsei University, College of Medicine, Seoul, South Korea; 4 Department of Surgery, Severance Hospital, Yonsei University, College of Medicine, Seoul, South Korea; 5 Department of Internal Medicine, Severance Hospital, Yonsei University, College of Medicine, Seoul, South Korea; Kaplan Medical Center, ISRAEL

## Abstract

**Purpose:**

To investigate whether a computer-aided diagnosis (CAD) program developed using the deep learning convolutional neural network (CNN) on neck US images can predict the BRAF^V600E^ mutation in thyroid cancer.

**Methods:**

469 thyroid cancers in 469 patients were included in this retrospective study. A CAD program recently developed using the deep CNN provided risks of malignancy (0–100%) as well as binary results (cancer or not). Using the CAD program, we calculated the risk of malignancy based on a US image of each thyroid nodule (CAD value). Univariate and multivariate logistic regression analyses were performed including patient demographics, the American College of Radiology (ACR) Thyroid Imaging, Reporting and Data System (TIRADS) categories and risks of malignancy calculated through CAD to identify independent predictive factors for the BRAF^V600E^ mutation in thyroid cancer. The predictive power of the CAD value and final multivariable model for the BRAF^V600E^ mutation in thyroid cancer were measured using the area under the receiver operating characteristic (ROC) curves.

**Results:**

In this study, 380 (81%) patients were positive and 89 (19%) patients were negative for the BRAF^V600E^ mutation. On multivariate analysis, older age (OR = 1.025, p = 0.018), smaller size (OR = 0.963, p = 0.006), and higher CAD value (OR = 1.016, p = 0.004) were significantly associated with the BRAF^V600E^ mutation. The CAD value yielded an AUC of 0.646 (95% CI: 0.576, 0.716) for predicting the BRAF^V600E^ mutation, while the multivariable model yielded an AUC of 0.706 (95% CI: 0.576, 0.716). The multivariable model showed significantly better performance than the CAD value alone (p = 0.004).

**Conclusion:**

Deep learning-based CAD for thyroid US can help us predict the BRAF^V600E^ mutation in thyroid cancer. More multi-center studies with more cases are needed to further validate our study results.

## Introduction

The BRAF^V600E^ mutation is the most commonly detected oncogene in thyroid cancer and is highly specific for papillary thyroid cancer (PTC) [1, 2]. Because of this high specificity, the mutation has been used in diagnostic methods adjunctive to fine needle aspiration (FNA) for thyroid nodules, especially those with indeterminate cytology results [1–5]. Also, the BRAF^V600E^ mutation is a known predictor of aggressive PTCs as it has been associated with higher cancer stage and a higher rate of extrathyroidal extension and lymph node metastases [6–8]. Considering that ultrasonography (US) features have also been associated with the BRAF^V600E^ mutation [3–5, 9], we can assume that the mutation test is a cost-effective tool for thyroid nodules with suspicious US features. However, US itself is inherently limited by its subjectivity, leading to the low reproducibility of its results [10, 11].

Artificial intelligence (AI) is being widely studied in the medical field with various applications being thought feasible in actual healthcare systems [12]. When diagnosing thyroid nodules on US, AI has shown comparable diagnostic accuracies to radiologists, with publications reporting the accuracies to be from 83% to 98% [13–15]. Compared to how diagnostic performances can vary according to the operator’s level of experience, AI can provide more objective results for US.

Several previous studies have focused on US features to predict BRAF status, and these studies have found that US characteristics associated with malignancy (marked hypoechogenicity, taller-than-wide shape, etc.) are also associated with BRAF positivity [9, 16, 17]. However, to the best of our knowledge, no studies have associated the BRAF^V600E^ mutation with US features using a computer-aided diagnosis (CAD) program. Therefore, we investigated whether a CAD program that was recently developed using the deep convolutional neural network (CNN) to diagnose thyroid cancer from US images of thyroid nodules could also help predict the BRAF^V600E^ mutation in thyroid nodules.

## Materials & methods

This retrospective study was approved by the Severance Hospital Institutional Review Board, with a waiver for patient consent (Approval number: 4-2019-1223). Signed informed consent was obtained from all patients prior to biopsy or surgical procedures.

### Patients

We collected patient data from Feburary 2019 to September 2019, during which 1817 patients were referred to our institution for thyroid surgery after undergoing biopsies at outside clinics and preoperative staging US at our institution, a tertiary referral center. Among them, 1348 patients were excluded because they were younger than 19 years old (n = 8), had nodules smaller than 10mm on US (n = 993), did not proceed with surgery (n = 230), had a final pathology of benignity (n = 87), and did not have the BRAF test performed on a pathologic specimen (n = 30). Finally, 469 thyroid cancers in 469 patients were included. Mean age of the patients was 42.3 years ± 12.8 (range, 19–86 years). Mean size of the tumors was 16.9 mm ± 8.3 (range, 10–62 mm). There were 353 (75.3%) women and 116 (24.7%) men.

### US examinations

Preoperative staging US was performed with a 5–12 MHz linear array transducer (iU22; Philips Medical Systems or EPIQ 7; Philips Medical Systems, Bothell, WA, USA) by one of nine radiologists specializing in thyroid imaging. All physicians were informed of previous cytology results and the location of the index lesion that underwent FNA or biopsy at outside clinics. Since June 2012, the physicians in our institution who perform staging US have filled out a report format for index nodules that includes nodule size and US features such as composition (solid, predominantly cyst, predominantly solid, spongiform, cyst), echogenicity (hyper-, iso-, hypo- and marked hypoechoic), margin (well, microlobulated, irregular), shape (parallel, not parallel) and calcification (eggshell, micro-, macro- and mixed calcification) [18]. One radiologist (K.J.Y.) who had 19 years of experience in thyroid imaging re-assigned the nodules according to the American College of Radiology (ACR) Thyroid Imaging, Reporting and Data System (TIRADS) using the prospectively recorded US features. Suspicious lymph nodes on US were also evaluated at the time of staging US. Finally, clinical T and N stages were reported according to the 8th American Joint Committee on Cancer (AJCC) cancer staging system [19].

### Image acquisition and CNN evaluation

One radiologist (K.J.Y) who had 19 years of experience in thyroid imaging reviewed the preoperative US images, selected a representative image for each thyroid tumor, saved it as a JPEG file in the picture archiving and communication system, and drew square region-of-interests (ROIs) to cover the whole nodule using the Microsoft Paint program (version 6.1; Microsoft Corporation, Redmond, WA, USA).

**Fig 1:**
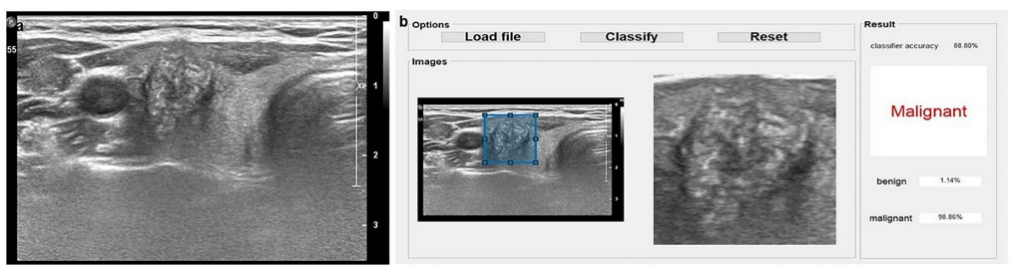
A 33-year-old woman with a thyroid nodule confirmed as suspicious for malignancy on US guided-fine needle aspiration (US-FNA).

**Fig 2:**
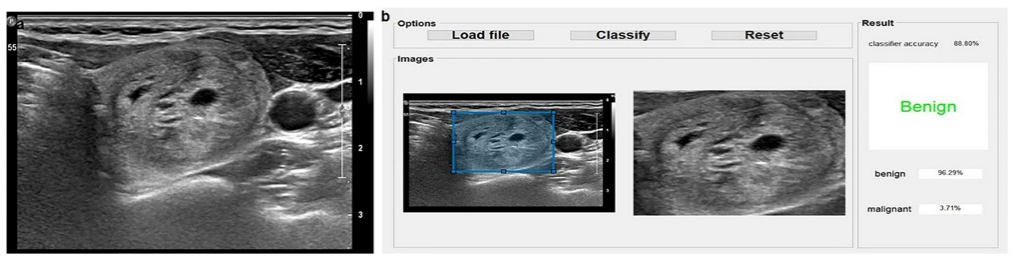
A 40-year-old woman with a thyroid nodule confirmed as suspicious for malignancy on US-FNA.

Recently, we developed a CAD program to diagnose thyroid cancer on US from 13,560 US images of thyroid nodules using the deep CNN (see S1 File for details on the CAD program). The CAD program provides risks of malignancy (0–100%) as well as binary results (cancer or not). Using the CAD program, we calculated the risks of malignancy from US images of the thyroid nodules (CAD value) (Figs 1 and 2).

US showed a 13 mm-sized solid, hypoechoic, and taller-than-wide shaped nodule with lobulated margin and punctate echogenic foci, which was categorized as “highly suspicious” on the American College of Radiology Thyroid Imaging Reporting and Data System (ACR TIRADS) **(a)**. The computer-aided diagnosis (CAD) program calculated the risk of malignancy as 98.86% (CAD value). **(b)**. The patient underwent total thyroidectomy and final pathology was conventional papillary thyroid carcinoma. Pyrosequencing was positive for the BRAF^V600E^ mutation.

US shows a 34 mm-sized predominantly solid, hypoechoic, and wider-than-tall shaped nodule with smooth margin and without echogenic foci, which was categorized as “mildly suspicious” on the American College of Radiology Thyroid Imaging Reporting and Data System (ACR TIRADS). **(a)**. The computer-aided diagnosis (CAD) program calculated the risk of malignancy as 3.71% (CAD value) **(b)**. The patient underwent left lobectomy and the final pathology was the follicular variant of papillary thyroid carcinoma. Pyrosequencing was negative for the BRAF^V600E^ mutation.

### BRAF^V600E^ mutation analysis

BRAF V600E mutants were evaluated using pyrosequencing per the manufacturer’s instructions. PCR amplification was performed with a forward primer (5′- biotin- TTCTTCATGAAGACCTCACAGTAA-3′) and a reverse primer (5′- CCAGACAACTGTTCAAACTGATG-3′) on a C1000 thermal cycler (BIO-RAD, California, USA). The pyrosequencing reaction was performed with a sequencing primer (5′- GGACCCACTCCCATCGAGATTT-3′) on a Pyromark Q24 instrument (Qiagen). The produced pyrogram was analyzed with the PyroMark Q24 software (Qiagen) to distinguish mutant versus wild-type alleles by relative peak height.

### Statistical analysis

We compared the demographics, ACR TIRADS categories and risks of malignancy from CAD between patients with and without the BRAF^V600E^ mutation using the independent two-sample t-test for continuous data and the Chi-square test or Fisher’s exact test for categorical data. Univariate and multivariate logistic regression analyses were performed to identify independent factors for predicting the BRAF^V600E^ mutation in thyroid cancer. As the ACR TIRADS and CAD values were assessed from the US images of thyroid nodules which could be correlated with each other, multicollinearity between the ACR TIRADS and CAD values was assessed with variance inflation factors (VIF). Multicollinearity was considered high when the VIF was > 10.

The multivariable model was only constructed with factors independently associated with the BRAF^V600E^ mutation. The predictive power of the CAD value and the multivariable model for the BRAF^V600E^ mutation in thyroid cancer were measured and compared with the area under the receiver operating characteristic (ROC) curves. The optimal cutoff for the CAD value to predict the BRAF^V600E^ mutation was identified using the Youden index. The performances of the CAD value and the multivariable model were compared using Delong’s method. All statistical analyses in this study were performed with SPSS statistical software (SPSS for Windows, version 25.0; IBM Corporation, Armonk, NY) and R (version 4.0.2.; R Foundation for Statistical Computing, Vienna, Austria). P-values of less than 0.05 were considered to indicate statistical significance.

## Results

In this study, 380 (81%) patients were positive and 89 (19%) patients were negative for the BRAF^V600E^ mutation. There were 422 (90%) conventional papillary thyroid carcinomas (PTCs), 26 (5.6%) follicular variant PTC, 9 (1.9%) diffuse sclerosing variant PTCs, 4 (0.9%) solid variant PTCs, 2 (0.4%) tall cell variant PTCs, 2 (0.4%) oncocytic variant PTCs, 1 (0.2%) Hobnail variant PTC, 1 (0.2%) Warthin-like variant PTC, 1 (0.2%) minimally invasive follicular carcinoma, and 1 (0.2%) medullary carcinoma.

Table 1 summarizes patient demographics and clinical characteristics according to the BRAF^V600E^ mutation. Patients with the BRAF^V600E^ mutation were older and had tumors of smaller size, higher ACR TIRADS scores, and higher CAD values than ones without the mutation.

**Table 1:** Comparison of patient demographics, ACR TIRADS categories and CAD values according to the BRAF^V600E^ mutation status.

	BRAF + (n = 380)	BRAF- (n = 89)	P-value
Age (years)	43.1 ± 12.9	38.9 ± 11.8	.005
Sex			.588
Female	288 (75.2)	65 (88.6)	
Male	92 (24.8)	24 (11.4)	
Tumor size (mm)	16 ± 7.3	20.8 ± 10.6	< .001
ACR TIRADS*			< .001
2	1 (0.3)	4 (4.5)	
3	6 (1.6)	11 (12.4)	
4	65 (17.1)	12 (13.4)	
5	308 (81)	62 (69.7)	
CAD value	77.9 ± 21.8	60.9 ± 31.6	< .001

*Fisher’s exact test

Note—Age, tumor size, and CAD value are shown as means and standard deviations.

Categorical variables are shown as numbers of patients with percentages in parentheses.

BRAF+ = BRAF^V600E^ mutation-positive, BRAF- = BRAF^V600E^ mutation-negative

ACR TIRADS = the American College of Radiology Thyroid Imaging, Reporting and Data System

CAD value = risk of malignancy from CAD

To determine potential predictors directly related to the BRAF^V600E^ mutation, univariate and multivariate logistic regression were performed. On univariate analysis, the BRAF^V600E^ mutation was associated with older age, smaller size, higher ACR TIRADS scores, and higher CAD values (Table 2). Multivariate logistic regression analysis was performed to assess the independent associations of the BRAF^V600E^ mutation with clinical factors. As the ACR TIRADS and CAD values did not show multicollinearity in the model (VIF was 1.366), we used both parameters in the regression model. On multivariate analysis, older age, smaller size, and higher CAD values were significantly associated with the BRAF^V600E^ mutation (Table 2).

**Table 2:** Univariate and multivariate logistic regression analysis for clinical factors based on the presence or absence of the BRAF^V600E^ mutation in thyroid carcinoma patients.

	Univariate			Multivariate		
	OR	95% CI	P-value	OR	95% CI	P-value
Age	1.029	1.008, 1.049	.005	1.025	1.004, 1.047	.018
Size	.944	.920, .967	< .001	.963	0.937, 0.989	.006
Men	1.156	.685, 1.951	.588	1.264	0.723, 2.211	.412
ACR TIRADS	2.109	1.491, 2.985	< .001	1.468	0.951, 2.266	.083
CAD value	1.025	1.016, 1.034	< .001	1.016	1.002, 1.027	.004

OR: Odds ratio, 95% CI: 95% confidence interval

ACR TIRADS = the American College of Radiology Thyroid Imaging, Reporting and Data System

CAD value = risk of malignancy from CAD

**Fig 3:**
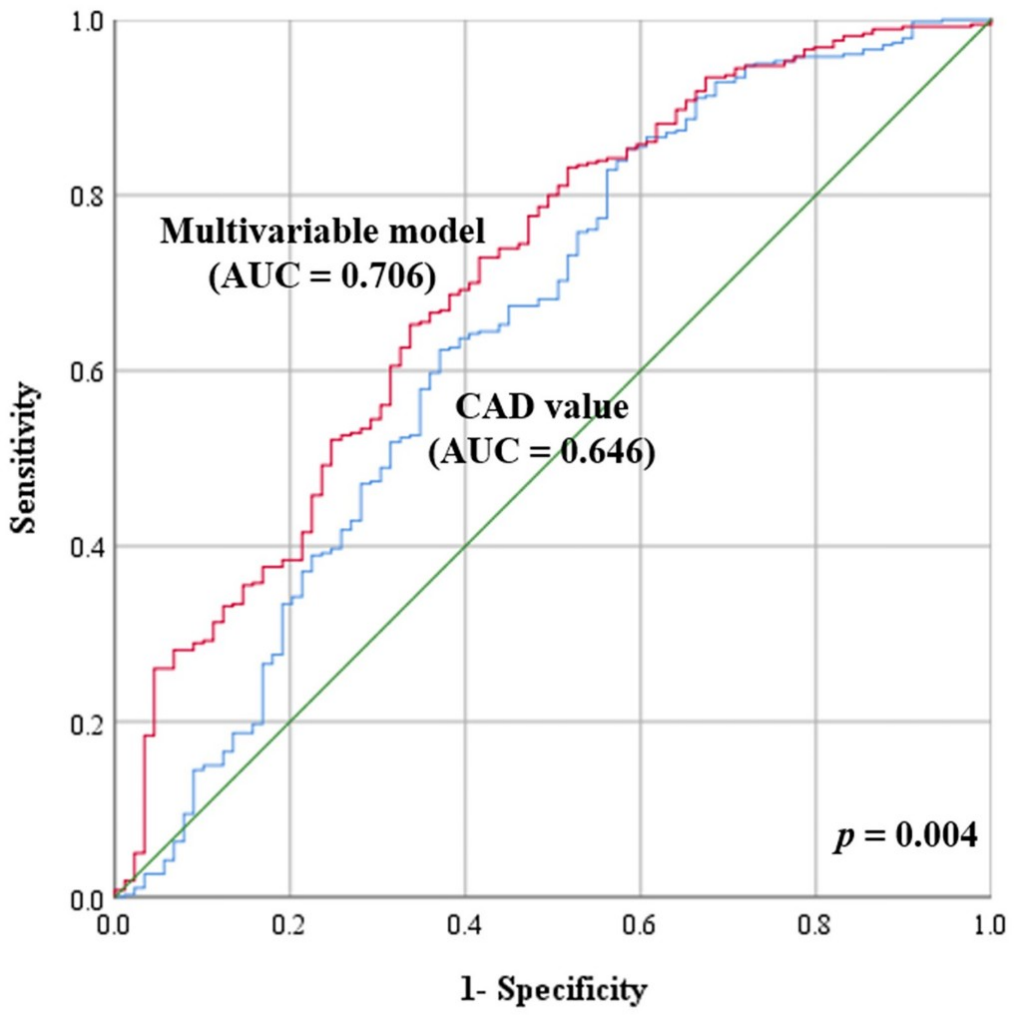
Receiver operating characteristic curves of the CAD value and the multivariable logistic model for predicting the BRAF^V600E^ mutation.

The multivariable logistic model was constructed using age, size, and the CAD value to predict the BRAF^V600E^ mutation. The ROC curves of the CAD value and the multivariable model were plotted to show the performances of the CAD value and the multivariable model for predicting the BRAF^V600E^ mutation in thyroid cancer patients. The CAD value yielded an AUC of 0.646 (95% CI: 0.576, 0.716) for predicting the BRAF^V600E^ mutation. The cutoff for the CAD value to obtain maximum accuracy for the diagnosis of the BRAF^V600E^ mutation was 57.7. Sensitivity, specificity, positive predictive value (PPV), and negative predictive value (NPV) of the CAD value for predicting the BRAF^V600E^ mutation was 85.3%, 41.6%, 59.4%, and 73.9%, respectively. The multivariable model yielded an AUC of 0.706 (95% CI: 0.644, 0.767) for predicting the BRAF^V600E^ mutation. The multivariable model showed significantly better performance than the CAD value only (p = 0.004) (Fig 3).

The CAD value yielded an AUC of 0.646 (95% CI: 0.576, 0.716) for predicting the BRAF^V600E^ mutation, while the multivariable model yielded an AUC of 0.706 (95% CI: 0.576, 0.716). The multivariable model showed significantly better performance than the CAD value only (p = 0.004).

## Discussion

The prevalence of the BRAF mutation in PTC ranges from 29% to 83% and almost all of the mutations are the BRAF^V600E^ form [20]. As the BRAF^V600E^ mutation is highly specific for PTC [21], a combination of cytology and the BRAF^V600E^ mutation has been considered a good diagnostic approach for thyroid nodules with indeterminate results on cytology [1]. Furthermore, the BRAF^V600E^ mutation is a known predictor of aggressive PTC; previous studies have reported that thyroid malignancies with the BRAF^V600E^ mutation present with higher cancer stage and higher rate of extrathyroidal extension and lymph node metastases than those without the mutation [6–8]. As the BRAF^V600E^ mutation can be used in both diagnosing thyroid cancer and predicting its aggressiveness, preoperative detection of the mutation may also help optimize the initial operative approach in patients with thyroid nodules [22–24]. However, the mutation analysis requires invasive procedures such as biopsy or surgical resection to retrieve specimens.

Several previous studies have tried to identify US features that can predict BRAF status [9, 16, 17]. A recent review article concluded that US features of PTCs correlate with their particular molecular mutations, and BRAF^V600E^ mutation is known to be associated with suspicious US findings, such as hypoechogenicity, non-parallel orientation, taller-than-wide shape, spiculated/microlobulated margins, and the presence of microcalcifications [25]. However, interpretation of US image is operator-dependent and inter-observer variability is moderate to substantial [10, 11, 26]. Radiomics is one of the emerging methods for predicting molecular characteristics of tumors, using quantitative imaging features extracted using data-characterization algorithms [27, 28]. A recent study evaluated the value of US-based radiomics for predicting BRAF^V600E^ mutation in pathologically proven PTCs, but radiomics features extracted from US had limited value [29].

In our study, the ACR TIRADS category assessed by radiologists was associated with the BRAF^V600E^ mutation in univariate analysis, but the correlation disappeared in multivariable analysis. In contrast, the CAD value, which was calculated from a program that we developed using deep CNN, was associated with the BRAF^V600E^ mutation in multivariable analysis. Our findings with the AUCs of the ROC curve indicate that the CAD value can help predict the BRAF^V600E^ mutation in thyroid cancer. Furthermore, the multivariable model which was composed with patient age, tumor size, and the CAD value showed significantly increased predictability. Considering that a deep learning algorithm allows consistent prediction, we can avoid or even overcome problems arising from inter-observer variability in US evaluations with the CAD value [30]. Therefore, the CAD program used in this study is expected to be a non-invasive and objective biomarker for prediction of the BRAF^V600E^ mutation in PTCs.

A number of CAD algorithms have been developed and implemented for the accurate diagnosis of disease, including binary logistic regression, support vector machines, and artificial neural networks [31]. Among them, deep learning with CNN has recently gained attention for its high performance in image recognition, and other researchers have developed deep learning CNN models by using radiological images for lesion detection, lesion evaluation, estimation of patient survival, etc. [32]. Many CNN models have already benefited clinical practice and this is especially so for thyroid malignancies, as deep learning CNN models developed for the detection, segmentation, and classification of thyroid nodules using neck US images have shown good performances [13, 14, 33–36]. A recent study reported that a deep learning CNN model developed to discriminate the BRAF^V600E^ and RAS mutations of PTCs from histological images showed 95% accurate predictions [37]. However, ours is the first study using deep learning-based CAD to predict the BRAF^V600E^ mutation in patients diagnosed with thyroid cancer. Previous studies reported associations between suspicious US features (such as marked hypoechogenicity and taller-than-wide shape) and the BRAF mutation, but most of these features showed weak correlation and the study results were inconsistent, probably due to intra- and inter-observer variability [9, 10, 16, 17]. As the CAD program provides objective probabilities of malignancy, it can potentially help diagnose BRAF^V600E^ mutations with more accuracy.

There are several limitations to this study. First, our study was of retrospective design and patient data were collected from a single tertiary referral center, which means a selection bias was inevitable. Second, an experienced radiologist retrospectively re-assigned categories to thyroid nodules according to ACR TIRADS using US images prospectively recorded by 9 radiologists. The re-assigned categories might not have fully reflected the prospectively recorded US features. Third, the correlation between the CAD value and cancer prognosis could not be confirmed due to the lack of a follow-up period. Further studies are needed to confirm whether the CAD value can be a predictor of cancer prognosis. Last, the prevalence of BRAF^V600E^ mutations among PTCs differs from country to country. The Korean population is known for its high mutation prevalence and 81% of the PTCs in our study showed the BRAF^V600E^ mutation [38]. Results might differ when the same study is conducted on populations from other countries.

In conclusion, the CAD program developed with deep learning can help predict the BRAF^V600E^ mutation in thyroid cancer. More multi-center studies with more cases are needed to further validate our study results.

## Supporting information

S1 File Computer-aided diagnosis (CAD) program using the convolutional neural network (CNN). (DOCX)

## Abbreviations

ACR: American College of Radiology
AI: Artificial intelligence
AJCC: American Joint Committee on Cancer
CAD: computer-aided diagnosis
CNN: convolutional neural network
FNA: fine needle aspiration
PTC: papillary thyroid cancer
ROI: region-of-interest
TIRADS: Thyroid Imaging, Reporting and Data System
US: ultrasonography
VIF: variance inflation factors
