# Supplementary material for: Artificial intelligence to predict the BRAFV600E mutation in patients with thyroid cancer
Source: PLoS One. 2020 Nov 25;15(11):e0242806. doi: 10.1371/journal.pone.0242806 (PMC7688114; doi:10.1371/journal.pone.0242806)
Supplement: S1 File — (DOCX) [file pone.0242806.s001.docx]

**S1 File. Computer-aided diagnosis (CAD) program using the convolutional neural network (CNN)**

A good deep learning process demands big data which is why transfer learning using pre-trained CNNs with a huge number of non-medical images is often used in image analysis. Our CAD algorithm utilizes transfer learning and goes through a fine-tuning process using thyroid US images (in which fine-tuning adjusts the weights of the pre-trained network by repeating backpropagation). The used pre-trained networks were AlexNet [1], GoogLeNet [2], SqueezeNet [3], and InceptionResNetv2 [4]. To train the algorithm, a total of 13,560 US images of either surgically confirmed or cytologic proven thyroid nodules from Severance Hospital were used. Another 634 US images of thyroid nodules, which had never been used in training, were used to test the performance of the algorithm and obtain corresponding AUC values. Once we obtained the AUC and probability values from each of the pre-trained CNNs (AlexNet, GoogLeNet, SqueezeNet, InceptionResNetv2), the final diagnosis was determined by the weighted average of the four probability values, where the weights were calculated from the AUC values. Matlab 2018b was used to build the following GUI (graphical user interface, see Figure 1 and Figure 2). GUI runs

1. load file – the user can load the image
2. classify – the user draws a box by clicking and dragging the cursor to show a ROI on the right hand side of the screen and the expected diagnosis with probability values
3. reset – it clears the images and the uploaded result.

**References**

1. Krizhevsky, A., Sutskever, I. & Hinton, G. E. ImageNet classification with deep convolutional neural networks. 2012:1097–1105 (Curran Associates Inc.).

2 Szegedy, C. et al. Going deeper with convolutions. Proceedings of the IEEE conference on computer vision and pattern recognition. 2015:1-9.

3. Iandola, FN. et al. SqueezeNet: AlexNet-level accuracy with 50x fewer parameters and<0.5 MB model size. arXiv preprint arXiv. 2016;1602.07360.

4. Szegedy, C., Ioffe, S., Vanhoucke, V., Alemi, AA. Inception-v4, inception-resnet and the impact of residual connections on learning. Thirty-First AAAI Conference on Artificial Intelligence. 2017.
